# Integrating Environmental Monitoring and Mosquito Surveillance to Predict Vector-borne Disease: Prospective Forecasts of a West Nile Virus Outbreak

**DOI:** 10.1371/currents.outbreaks.90e80717c4e67e1a830f17feeaaf85de

**Published:** 2017-05-23

**Authors:** Justin K. Davis, Geoffrey Vincent, Michael B. Hildreth, Lon Kightlinger, Christopher Carlson, Michael C. Wimberly

**Affiliations:** South Dakota State University; South Dakota State University; South Dakota State University; South Dakota Department of Health, Pierre, SD, USA; South Dakota Department of Health; South Dakota State University

## Abstract

**Introduction:**

Predicting the timing and locations of future mosquito-borne disease outbreaks has the potential to improve the targeting of mosquito control and disease prevention efforts. Here, we present and evaluate prospective forecasts made prior to and during the 2016 West Nile virus (WNV) season in South Dakota, a hotspot for human WNV transmission in the United States.

**Methods:**

We used a county-level logistic regression model to predict the weekly probability of human WNV case occurrence as a function of temperature, precipitation, and an index of mosquito infection status. The model was specified and fitted using historical data from 2004-2015 and was applied in 2016 to make short-term forecasts of human WNV cases in the upcoming week as well as whole-year forecasts of WNV cases throughout the entire transmission season. These predictions were evaluated at the end of the 2016 WNV season by comparing them with spatial and temporal patterns of the human cases that occurred.

**Results:**

There was an outbreak of WNV in 2016, with a total of 167 human cases compared to only 40 in 2015. Model results were generally accurate, with an AUC of 0.856 for short-term predictions. Early-season temperature data were sufficient to predict an earlier-than-normal start to the WNV season and an above-average number of cases, but underestimated the overall case burden. Model predictions improved throughout the season as more mosquito infection data were obtained, and by the end of July the model provided a close estimate of the overall magnitude of the outbreak.

**Conclusions:**

An integrated model that included meteorological variables as well as a mosquito infection index as predictor variables accurately predicted the resurgence of WNV in South Dakota in 2016. Key areas for future research include refining the model to improve predictive skill and developing strategies to link forecasts with specific mosquito control and disease prevention activities.

## Introduction

The mosquito-borne West Nile virus (WNV) has caused more than 43,937 human cases reported to the CDC in North America since its first detection in New York State in 1999 1. Human WNV disease burden is high in the Great Plains region of the United States, particularly in South Dakota (SD). From 2004-2015, a total of 1,084 cases were reported for SD, representing an estimated incidence rate of 133 cases per 100,000 compared to 10 per 100,000 for the entire US 2. Reported case counts in SD vary substantially among years – from more than a thousand in the initial epidemic year 2003 to just two cases in 2011. The ability to predict human cases in advance would permit public health and mosquito control officials to allocate resources more efficiently and inform the public of its actual risk, without the risk of "message fatigue" associated with constant warnings of high danger.

Yearly variability in human cases partly reflects variations in climate and their influences on the virus, the mosquito vectors, the avian hosts, and their interactions. The dynamics of South Dakota’s primary WNV vector (*Culex tarsalis* Coquillett) have been related to seasonal weather patterns and interannual climate variability 3 ^, ^4^, ^5. Human cases have also been associated with precipitation 6^, ^7^, ^8 and temperature 8^, ^9^, ^10^, ^11^, ^12. Gridded meteorological datasets are especially suitable for predictive efforts, as they provide complete geographic coverage, are freely accessible via online archives, and have been shown to correlate with human cases 13^, ^14.

However, epidemiological feedbacks affecting host population dynamics and immunity cycles also influence WNV transmission among birds and the consequent risk to humans, and these may not be explicable in terms of meteorological data alone 15. As a result, more direct observations of the virus in the system, such as mosquito infection rates, are important for predicting human disease cases 16^, ^17^, ^18. These data are not solutions in themselves; Kwan et al. note that both meteorological and biological indices are critical in describing risk to human populations and point to instances in which one or another indicator fails when used alone as a predictor 15.

Additionally, most time-series models of disease are retrospective even if theoretically predictive. While there has been broad interest in applying predictive models to forecast outbreaks of mosquito-borne disease 19^, ^20^, ^21, genuinely *a priori* predictions of mosquito-borne illness are relatively rare in the scientific literature. Some examples include predictions of dengue before the 2014 FIFA World Cup Brazil 22^, ^23, dengue imported by air travel in Europe 24, Chikungunya in Europe 25, WNV in the US in early 2014 and 2015 9, and Zika in the US 26. More common are studies that assess the relationships among cases and covariates without making predictions of the future. As illustrated by the review of Zinszer et al. of malaria models, most evaluations of model predictions assess goodness-of-fit with already-available data, which may have even been used to fit the model 21. As a result, the body of retrospective studies contains many models that have never been tested against independent data, and are likely overfit. To critically evaluate the potential of disease forecasting models, there is a need for researchers to develop models, use them to make prospective forecasts, and then evaluate these predictions using independent observations that are collected *post hoc*.

The objective of this study was to evaluate a predictive model of West Nile virus in South Dakota (SD), which was used to make whole-year forecasts at the beginning of the 2016 WNV season, as well as short-term weekly forecasts throughout the season. This modeling approach was unique in that it incorporated not only weather data but also WNV infection data to predict spatial and temporal patterns of WNV cases. Because the model was specified and parameterized prior to the start of the 2016 WNV season, this exercise provided an opportunity to make *a priori* predictions and then validate them using independent data. The model’s estimates were generally accurate for 2004-2015, as were its predictions for the 2016 WNV season. In particular, warmer weather in early 2016 indicated that the WNV season would begin earlier than usual, and high mosquito infection rates detected early in the WNV season provided further evidence to support predictions of a high WNV human case burden.

## Materials and Methods

**Data sources**

Human case data: Records for 1,249 human WNV cases from 2004 to 2015 were provided by the South Dakota Department of Health (SDDOH). While there were 1,076 human cases in 2002-2003 alone, the large number of cases in these initial epidemic years reflected the unique conditions when WNV was first introduced into the region 27. As a result, these atypical years were excluded and we focused on endemic WNV from 2004 to the present.

All disease cases were laboratory-confirmed and satisfied the appropriate criteria of the CDC definitions; it should be noted that case definitions were updated during the study period 28. We do not have data on the specific techniques used to confirm diagnoses, as these data were collected by the SDDOH from reporting clinicians and various levels of detail are associated with each case. Most cases were likely confirmed by ELISA, and other methods such as plaque-reduction neutralization tests (PRNTs) may have also been used 29. All reported viremic blood donors were also included in the set although they would not typically be called cases of disease because they are usually asymptomatic. Blood donation facilities sent samples to out-of-state labs for nucleic acid amplification tests (NATs) and the SDDOH received reports of positive tests 30.

We summarized the total number of cases in each county in each week. Each record contained the person’s county of residence and the date on which the patient began showing symptoms or, for viremic blood donors, the date when the blood was donated. These data were obtained through a formal data-sharing agreement between the SDDOH and South Dakota State University. The project was determined to be exempt from IRB review because the human case data are collected as a normal part of the SDDOH’s surveillance process and do not contain any personally identifying information.

For every combination of county (66 total) and week in 2004-2015, beginning with the week Jan. 5-11, 2004, a 0/1 variable indicated whether the county reported no disease cases or viremic donors that week (0) or had at least one disease case or viremic donor (1). The county-week was chosen as the object of study, rather than the number of cases per county per week, because most county-weeks had zero cases and positive county-weeks usually had just one case.

*Weather data*: Raw hourly mean temperature (deg C) and total precipitation (mm) data were obtained for all dates beginning Jan. 1, 2003, from the North American Land Data Assimilation System (NLDAS) atmospheric forcing data 31. These data have a spatial resolution of one eighth-degree (approximately 10.7 km E-W by 13.9 km N-S at 40° north latitude) and were derived from the assimilation of data from multiple sources, including the North American Regional Reanalysis dataset and the US Climate Prediction Center unified gauge-based precipitation analysis. The NLDAS data were downloaded from the NASA Goddard Earth Science Data and Information Services Center (https://daac.gsfc.nasa.gov).

Temperature and precipitation were obtained for every combination of county and hour by sampling the appropriate NLDAS layer at each county centroid. These values were then aggregated to compute daily mean temperature and total daily precipitation for every combination of county and day. The object of study was the county-week combination, and each combination of county and epidemiological week was associated with 362 lagged variables. These variables included temperature and precipitation ranging from the day the week began to 180 days prior, so that every county-week had a 6-month daily meteorological history of temperature and precipitation.

The model of human disease described below relies on this 6-month history, in addition to mosquito infection data, to provide estimates for human WNV in any county-week. NLDAS data may lag up to four days before the present, and model estimates would be restricted to the current week if actual observations alone were used. To permit predictions into the future, every county-week in 2016 was assigned a 6-month daily "history," combining observed NLDAS data and projected future meteorological variables. Observed, historical data were used when possible. Otherwise, total precipitation in a county-day was projected as the mean total precipitation in that county on that day of the year in 2003-2015, and mean temperature was projected as the 55th percentile of all previous daily mean temperatures over 2003-2015. These choices were consistent with long-term climate forecasts for South Dakota, consulted in Spring 2016, which indicated average to slightly-above-average temperatures and average precipitation 32. Thus in Spring 2016, our only information about meteorological conditions during the WNV season was based on projections of historical data. As new NLDAS observations were obtained during each week of the WNV season, these projections were replaced by observed data.

We used the human disease model (described in the next section) to generate predictions based on these meteorological data; these can be viewed in two ways. First, *whole-year predictions* were made for every county-week in 2016, including weeks already in the past, using whatever NLDAS data were available when the predictions were made. For example, whole-year predictions made on July 5th relied on observed temperature and precipitation up to July 1st (constrained by the availability of the NLDAS), with projected climate variables from July 2nd to December 31st. Hence, every county-week in 2016 from January to December had the 6-month history of temperature and precipitation that the human disease model requires to make predictions of risk, but around half of the meteorological data for the year were projected. As the year progressed, new whole-year predictions were made each week and were influenced more by observed meteorological variables and less by projections.

Second, *short-term predictions* were the small subset of whole-year predictions made just one week into the future, using no more than two weeks of projected meteorological data. For example, short-term predictions made on July 5th used observed weather data up to July 1st, projected data up to July 12th, and included predictions for the 66 counties of SD in the week of July 12th - 18th. These were individual slices in time, each taken from the corresponding whole-year prediction (in this example, from the July 5th whole-year prediction). These short-term predictions were thus based on meteorological histories that were nearly complete when the predictions were made. From the standpoint of public health and mosquito control, this short-term view was considered most relevant for decision-making, and short-term predictions were used to generate weekly WNV risk maps (e.g. Figure 3 below).

*Mosquito infection data*: Mosquito infection data for 2004-2015 were obtained from 28 reporting counties in South Dakota (Figure 1), including state, county, city, and tribal entities operating both in concert and independently. On average, counties began submitting mosquitoes for testing on the 163rd day of the year (June 12th) and ended on the 238th (August 26th). All mosquitoes were collected with carbon dioxide-baited CDC light traps equipped with photo-switches and air-activated gates. Mosquitoes, identified by morphological characteristics by trained individuals within local agencies, were separated into single-species pools containing no more than 50 mosquitoes. Much of the mosquito testing was performed by the SDDOH, using the methods described by Lanciotti et al 33. Some mosquito testing was performed locally by individual mosquito control districts, with the RAMP test (ADAPCO) according to the manufacturer's recommendation. Only *Cx. tarsalis* data were used for statistical modeling.

**Study Area in Context:**
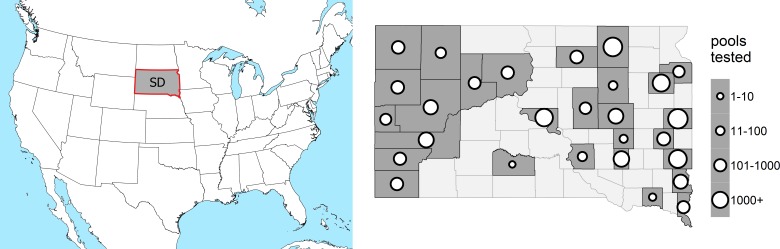
Location of South Dakota (left) and distribution of counties in South Dakota with number of pools sampled per county over 2004-2015 (right). Lighter counties did not submit pools of mosquitoes for testing.

**Statistical modeling**

*Model of mosquito infection rate*: All *Culex tarsalis* pools collected before July 31st were used for analysis; we call these early-season data, as only 18% of SD human cases have occurred historically before the end of July. A generalized linear mixed model was used to estimate the probability that a pool would test positive (pool = 1) as a function of the day of the year *d*. The glmer function (lme4 package, R x64 version 3.2.2) was used to conduct a fit on Bernoulli data (pool=0/1) with model

\begin{equation*}\operatorname{logit P}(\operatorname{pool} = 1 | d, \ \operatorname{year}) = \sum_{i=0}^{2} {\(c_i + z_i\) f_i(d)}\end{equation*}

where *c_i_* were constants, *z_i_* were *a priori* uncorrelated random normal effects with year as subject, and *f_0_, f_1_, f_2_* were functions of *d* resulting from applying the Gram-Schmidt algorithm to the three functions *p_i_(d)=d^i^, i* = 0, 1, 2, where *d* ranged from 1 (January 1st) to 212 (July 31st). That is, the (logit) rate of positive pools early in the year was modeled to rise smoothly as a quadratic curve, with each year differing slightly from a hypothetical average year.

The *mosquito infection threshold date* for every year was defined as the earliest day of the year (1-366) at which the point estimate for pool positive rate rose above 1%. All available data were used to estimate fixed threshold dates for every year in 2004-2015 and the estimated date for 2016 was updated every time new mosquito infection data became available. The threshold date was then used as one of the independent variables in the model of human infections, described below.

The primary virtue of this model is that it gives reasonable estimates even when few data are available – in this case, when few mosquito pools are available at the beginning of the WNV season. A simpler fixed effects model (lacking the *z_i_* terms) would, in the early season, indicate no risk of WNV for the subsequent year until at least one positive pool was reported. In contrast, the mixed-effects model estimates that a year will be average for WNV risk until the data indicate otherwise, and its estimates tend to respond gradually to new data. Hence, both weather and mosquito data were imputed when missing and both were always included in the model of human disease, but this additional modeling step was necessary to smooth and impute missing early-season mosquito infection rates, which are inherently more heterogeneous than the meteorological variables.

*Model of human infections*: A logistic regression was used to estimate the probability that a county would be positive in a given week. Covariates included county as a set of indicator variables, the mosquito infection threshold date defined above, and daily mean temperature and daily total precipitation, summarized by eighth-degree polynomial distributed lags from 180 days prior to the day the week began 34. Model specification was determined based on a fit conducted on human cases from 2004-2015 (glm function, stats package). The final model was specified in May 2016 prior to the start of the WNV season, and no data from 2016 were used in parameter estimation.

We assessed model performance by 1) evaluating model fit using the 2004-2015 historical data, 2) comparing short-term predictions for 2016 with observed human case data, and 3) comparing whole-year predictions for 2016 made on July 5th and July 29th with observed human case data 35^, ^36. July 5th was early enough in the year that few mosquito infection data would be available and meteorological data would be the main driver of model estimates. By July 29th, enough mosquito data have typically been received so that both components of the human disease model would contribute, but this was still early enough in the season that genuinely informative predictions could be made.

Although whole-year predictions cover January 1st - December 31st, model estimates were evaluated using human data only in July-September. There were cases in May – June (1.7% of total cases) and October – December (1.8%), but it is mostly trivial to predict that county-weeks in these months will tend to be negative. To evaluate fit, the area under the response-operator curve (or c-statistic) was calculated (roc and auc functions, AUC package) and Hosmer-Lemeshow goodness-of-fit test was conducted (hoslem.test function, ResourceSelection package).

## Results

*Mosquitoes*: In 2004-2015, 834,263 mosquitoes were submitted for WNV testing in 32,866 single-species pools. Most pools submitted for testing (79%) contained *Cx. tarsalis*. Of these, 1,013 (3.8%) were positive for the virus. Most of the positive pools (92%) were *Cx. tarsalis*. There were also positive pools of *Cx. pipiens* L. (2.4% of pools tested), *restuans* Theobald (3.1%), *salinarius* Coquillett (1.0%), and *Aedes vexans* Meigen (0.7%). None of the twelve other species tested were positive.

In 2016, 52,828 mosquitoes were tested in 2,341 pools, of which 94% contained only *Cx. tarsalis*. Of the *Cx. tarsalis* pools tested, 5.9% were positive. Fewer of other species were tested than in previous years because *Cx. tarsalis* is now assumed to be the primary vector in South Dakota. Two *Cx. pipiens* pools (0.1%) were also positive.

*Human cases (demographics)*: A total of 1,131 human WNV cases and 130 viremic blood donors from 2004-2015 were available for analysis, in 942 positive county-weeks; the majority of positive county-weeks represented single cases. Most (67%) were diagnosed on the basis of fever or other minor complaints, while 23% were neuroinvasive and 9% detected during blood donation. Provisionally final data published by the SDDOH on Dec 15th 2016 indicated that 2016 was an above-average WNV year. There were 167 disease cases and 16 viremic blood donors in 123 positive county-weeks, including 6 deaths. Consistent with other years, 10% were detected during blood donation and 23% had neuroinvasive disease 37.

*Human cases (fits to 2004-2015 data)*: The model for human cases achieved a c-statistic (or AUC) of 0.847 when tested on July-October data in 2004-2015; this implies that the model was generally able to discriminate between positive and negative county-weeks, and is considered excellent discrimination by Hosmer and Lemeshow 38. The model was not rejected by the Hosmer-Lemeshow goodness-of-fit test (p = 0.463, rejected if below 0.050); this result implies that estimated probabilities did not as a whole differ so much from observations that the model should have been rejected as poorly-calibrated.

The mosquito infection threshold date had an estimated coefficient of -0.0541 (SE: 0.0043). Every additional week for which the rate of positive pools remains below the 1% threshold, the odds of any county being positive in any week fall by a factor of 32% for the whole year. Estimated threshold dates ranged at their earliest from 165 (=June 14th) in 2012 to their latest at 205 (=July 24th) in 2011. These years were the most and least intense years in the set for human disease, with 245 and 2 cases respectively. The threshold date and total positive county-weeks by year in 2004-2015 correlated well, with Spearman rho = -0.76.

Estimates of county-specific constants are not reported here, but ranged from -2.0 to 3.4. The parameters of the distributed lags for temperature and precipitation are coefficients of eighth-degree polynomials and have no meanings individually. Rather, we reconstruct the estimated distributed lag dependence polynomials in Figure 2. For ease of comparison, both estimated distributed lags have been standardized, or divided by the standard deviation in daily temperature (12.9 deg C) and precipitation (0.22 mm), so that comparison of effect size is easier. Maximum dependence on temperature occurred 27 days before the week in question began. The relationship with precipitation was more ambiguous, and no clear pattern of dependence emerged.

**Estimated dependence functions:**
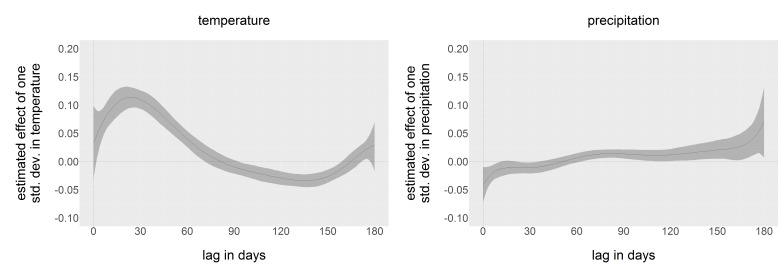
Dependence of county-week positivity on temperature and precipitation in the past six months, estimated by distributed lags. Estimates have been standardized by the standard deviation of temperature and precipitation, so that dependence on temperature and precipitation are directly comparable. 95% confidence intervals are in grey.

*Human cases (short-term predictions in 2016)*: Short-term predictions for 2016, made one week in advance and driven by the mosquito infection threshold date and observed weather data, were generally accurate, although the ability of the model to predict cases within individual weeks varied. Six weeks of short-term predictions from 2016 are displayed in Figure 3 to illustrate the evolution of estimated risk over the course of a heavy WNV year.

For the week beginning June 6th 2016, the c-statistic for the model’s predictions was 0.9672; three counties known for high case counts had predictable, early cases. The c-statistic fell to 0.734 for the week beginning July 18th, as cases began showing up elsewhere in the state. The discriminatory power of the model ranged from 0.65 to 1.00 in 2016 on a weekly basis but was acceptable (> 0.80) in August; i.e. the weekly spatial pattern of cases was predicted with acceptable accuracy at the height of the WNV season. When the model was evaluated over all county-weeks from July through September, the overall fit was good with c-statistic 0.856 and Hosmer-Lemeshow goodness-of-fit p = 0.1999.

**Examples of short-term, spatial predictions:**
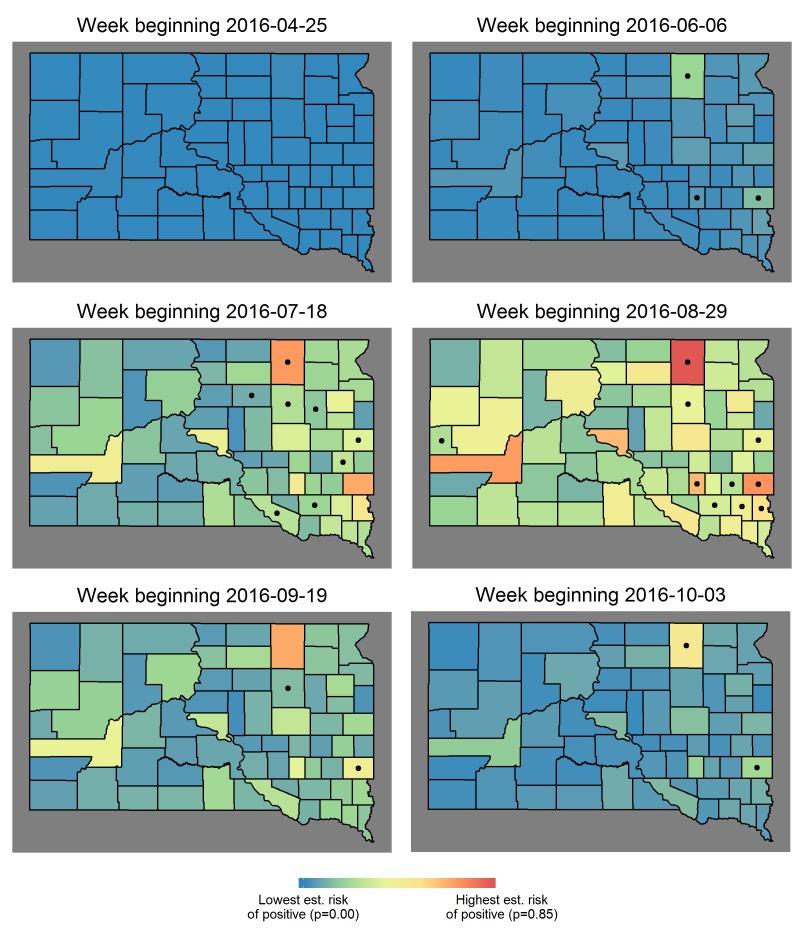
Six weekly estimates of human risk during 2016, an outbreak year in SD. Estimated weekly risk reached its highest point over the entire study period (represented by red), which occurred in early August 2012 and was nearly reached again in 2016. Dots indicate at least one human case was reported in that county, that week.

Summing all estimated probabilities in any week gives an estimate of total positive counties that week. Total positive counties per week for 2016, estimated and observed, are displayed in Figure 4. The observed positive counties fluctuated from week to week but generally followed the model predictions and were nearly all within the 95% prediction interval. Predicted and observed values for 2016 were both higher than the observations in 2015, but fell below the peaks observed in 2012, when the last major WNV outbreak in South Dakota occurred. Of special note is the early, rapid start to the 2016 season compared to other years, which was correctly predicted.

**Short-term, statewide predictions in 2016:**
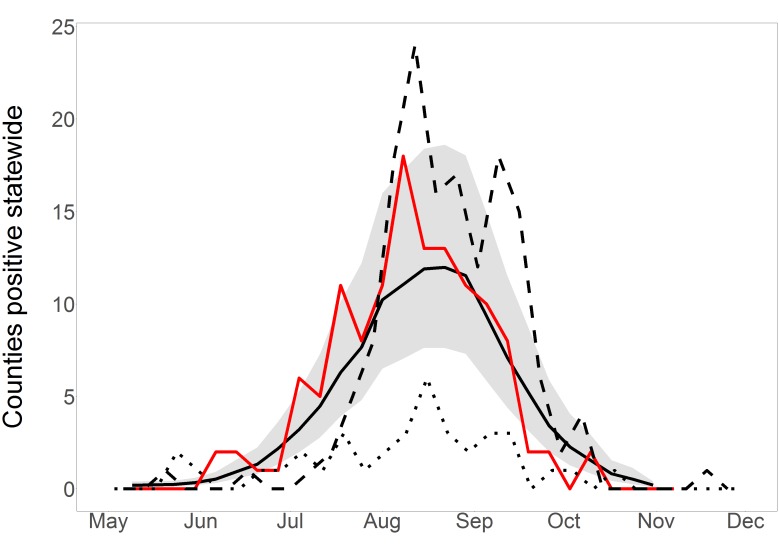
Statewide short-term predictions in 2016. Observed positive county-weeks, reported by the SDDOH in December, are in red. Model predictions are solid black with 95% prediction interval in grey. For comparison, 2012 is dashed and 2015 is dotted. Observed and predicted before May are both negligible and are omitted.

*Human cases (whole-year predictions made on July 5th)*: When we made whole-year predictions on this date, we had not yet received enough mosquito infection data to indicate that the mosquito infection threshold date for 2016 differed from average, but record-breaking high temperatures in early March and June drove the predictions of a higher- and earlier-than-average WNV year (Figure 5). We predicted that there would be 88 total positive county-weeks for 2016 (95% prediction interval 56-147), which placed 2016 between 2012 (177 positive county-weeks) and 2015 (42). The mean and median yearly total positive county-weeks for 2004-2015 were 78 and 53. That is, weather data alone indicated that 2016 would be an above-average year for WNV. Tested on final 2016 data, these predictions had c-statistic 0.836; whole-year predictions made at this time correctly discriminated between positive and negative county-weeks. Yet predictions failed the Hosmer-Lemeshow goodness-of-fit test with p = 0.0073; although the model predicted a high year, it still systematically underestimated risk and its early predictions were considered poorly calibrated.

*Human cases (whole-year predictions made on July 29th)*: By this date, we had received enough mosquito infection data to revise our estimated total positive county-weeks for 2016 to 116 (95% PI: 72-185), placing it fifth highest in the 13-year period 2004-2016 (Figure 5). This prediction was essentially stable until the end of the year, rising to 119 by October (95% PI: 77-195). On Dec. 15th 2016, at the end of WNV transmission season, the SDDOH reported 146 clinical cases and 16 viremic blood donors, for a total of 123 positive county-weeks, close to the 116 total predicted on July 29th and within the 95% prediction interval. When these predictions were tested on July-October 2016 data, discriminatory power was good (c = 0.855) and predictions were well-calibrated (p = 0.702). That is, the model discriminated between positive and negative county-weeks, and estimated risk had been raised to appropriate levels by the inclusion of mosquito infection data. Whole-year, statewide predictions on these two dates are displayed in Figure 5 to illustrate the evolution of predictions over the course of a year.

Notably, the case counts reported during the WNV season lagged unpredictably behind final case counts that were eventually obtained. For example, initial reports released by the SDDOH indicated that 6 cases had been diagnosed by July 5th but, when final data were received in December, it was learned that there had actually been 16 cases by this date. Similarly, an initial count indicated 16 cases before July 29th, but this was revised to 45 when final data were received in December. Because there was no clear relationship between in-the-moment and final case counts, case counts reported during the season were not used in any aspect of model fit or evaluation.

**Whole-year, statewide predictions in 2016:**
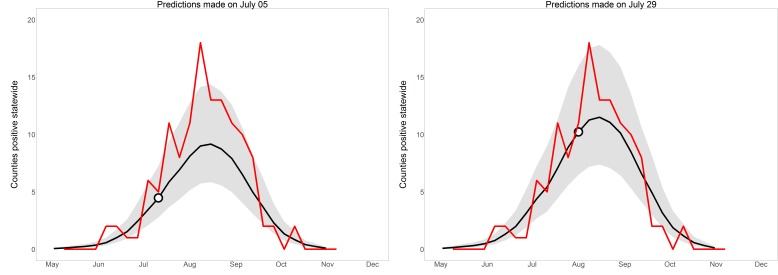
Statewide whole-year predictions in 2016, made on two dates early in the 2016 WNV season. Observed positive county-weeks, reported in December, are in red. Model predictions are in black, with 95% prediction intervals in grey. Dots indicate the slice of the whole-year predictions that contributed to the one-week-ahead, short-term predictions displayed in Figure 4; each whole-year prediction provided one such short-term view.

## Discussion

A statistical model utilizing entomological and meteorological data explained historical variations in human case reports and also yielded generally accurate predictions for the upcoming year. Model predictions made in early 2016 were based solely on meteorological data and correctly identified a higher-/earlier-than-average WNV year due to increased temperatures. However, before mosquito infection data became available the model underestimated the true intensity of WNV transmission in 2016. The inclusion of mosquito data in late July brought model estimates up to an appropriate level, which matched eventual case reports.

Other WNV modeling schemes have typically used either entomological indices 39 or meteorological risk factors 6^, ^40^, ^41, but we contend that real-time predictive models will need to integrate both types of information to better leverage their relative strengths and minimize their limitations. Gridded meteorological data, in this case from the NLDAS, tend to be complete over time and space, are publicly accessible, and have well-established relationships with a number of critical processes related to mosquito-borne disease transmission. Mosquito surveillance efforts typically have sparser and more uneven coverage, and the data are typically collected and compiled by municipal, county, and state public health and mosquito control agencies. Thus, it should be relatively feasible for these agencies to combine meteorological datasets with entomological surveillance systems to support integrated modeling of WNV risk.

The positive lagged relationship of cases with temperature, peaking near 4 weeks prior to the week in question, is in accord with previous research that has consistently found positive temperature effects over a range of temporal scales. Our results are most similar to those of Paz et al., who found strong correlations between WNV febrile cases and temperatures at 4 week lags in Russia and Romania 12. Soverow et al. found positive correlations between symptom onset and temperatures up to 4 weeks prior, in a model of human cases in 17 US states 42. Temperature associations with WNV reported in other studies tend to be longer-term, extending from 7 weeks to previous seasons 40^, ^43^, ^44. In contrast, previous studies of temperature effects on WNV vector abundance in South Dakota have found short-term lagged associations ranging from 1-2 weeks 39.

The relationship with precipitation, here and elsewhere, is less clear. Negative associations with WNV at lags of a week or less may indicate a short-term suppressive effect on mosquito activity, whereas positive effects at lags of more than two months may reflect long-term effects of increased breeding habitats on mosquito abundance. However, the magnitude of these effects is negligible compared to that of temperature. This finding is most consistent with the description of Hubalek, who associated outbreaks with flooding followed by extended periods of warm, dry weather 45. Soverow et al., on the contrary, found that recent, heavy precipitation events are associated with higher incidence 42. DeGroote et al. found relationships between precipitation and incidence that differed in direction depending on year on a national scale 41. Wimberly et al., focusing on the Northern Great Plains region of the US, found that relationships could be positive or negative depending on geographic location 40. Landesman et al. found that outbreaks were associated with precipitation in the previous year in a national model 6. This variety of results likely reflects the habitat preferences of different vector mosquito species, combined with the diversity of hydrological factors that control the development of temporary water bodies.

Mosquito infection data account for important aspects of the transmission cycle unrelated to meteorological conditions. Increased temperatures were indicative of higher risk in the coming year, but the model only indicated the true height of the season when data on mosquito infection status became available. A model of human risk based solely on meteorological data may suffice to indicate that a season will be above or below average. However, where these data on mosquito infection status are lacking, we suspect that important influences will remain invisible.

For example, Kwan et al. found that low levels of avian immunity during the preceding winter and spring put human populations at risk of WNV transmission 15. It is reasonable that our early-season mosquito infection data would shed light on the rate of amplification in the state’s avian hosts. However, the geographic pattern and intensity of mosquito surveillance efforts in SD has varied through the years, making it difficult to compare infection rates from year to year. Even in the study of Kilpatrick and Pape, in which 15 counties reported weekly data almost uniformly for five years, early-season mosquito infection rates could not be reliably estimated locally, and statewide proxies were sometimes better predictors of risk to human populations 39. In the current study, we were able to generate stable and consistent estimates from a very heterogeneous dataset by including a separate model of early-season mosquito infection rate. The threshold date that was derived from this model proved to be a powerful predictor of transmission in the upcoming WNV season.

The integrated modeling approach presented here has the potential to support public health and mosquito control activities aimed at reducing the burden of WNV and other mosquito-borne diseases. Combining information about mosquito infection rates with meteorological conditions allowed us to make effective predictions of WNV risk for the upcoming week, as well as an accurate whole-year estimate of the magnitude of expected transmission over the entire WNV season. We communicated our forecasts to the SDDOH and local municipalities in July 2016 and recommended that aggressive mosquito control activities be instituted, even though mosquito numbers were relatively low and few human cases had been reported at that time.

There are a number of opportunities to enhance the modeling approach to improve predictions in future years. The current county-level resolution is still relatively coarse, and predictions at the municipality or census block resolution would be desirable to more effectively target interventions. Modeling at this finer scale will necessitate identifying and mapping more specific, landscape-level risk factors for WNV rather than relying on county-level effects 46^, ^47. It may also be possible to improve the accuracy of the predictions by using different formulations of the independent variables, including different temperature indices (e.g. nighttime temperature summarized during the times when the vector mosquitoes are most active) and more direct estimates of mosquito breeding habitat than precipitation (e.g., direct estimates of surface water obtained from satellite remote sensing or soil moisture derived from hydrological models). Our two-step modeling approach could also be used to derive stable estimates of other entomological indices, such as the vector index, which incorporates mosquito abundance as well as infection rate.

Perhaps most importantly, there is a need to better connect these WNV forecasts with targeted public health responses that can reduce transmission and ultimately reduce the morbidity and mortality burden of WNV outbreaks like the one in 2016. An accurate, well-validated predictive model can help form the frontline of city, county, and tribal mosquito control programs, where this information can be used to more effectively target efforts to reduce vector populations, limit transmission, and prevent disease.

## Corresponding Author

Please contact Dr. Michael Wimberly (michael.wimberly@sdstate.edu).

## Data Availability

Daily meteorological and mosquito infection threshold data, used in the model of human disease, are available in CSV form from the Figshare repository 48. Human case data were obtained through a data-sharing agreement with the South Dakota Department of Health that does not allow redistribution of the data. To make a request for data access, contact the South Dakota Department of Health at 605-773-3737.

## Competing Interests

The authors have declared that no competing interests exist.
